# Spray Drying of Aonla Juice Mixed With Giloy Extract for Process Optimization, Product Development and Characterization

**DOI:** 10.1002/fsn3.72031

**Published:** 2026-06-19

**Authors:** Gurpreet Jhally, Sajeev Rattan Sharma, Kulwinder Kaur, Mohammad Shafiq Alam, Sunita Rani, Tarsem Chand Mittal, Prasad Chavan, Amit K. Jaiswal

**Affiliations:** ^1^ Department of Processing and Food Engineering Punjab Agricultural University Ludhiana India; ^2^ Department of Food Technology Dr Rajendra Prasad Central Agricultural University Pusa, Samastipur India; ^3^ Department of Chemistry, Faculty of Sciences Guru Kashi University Talwandi Sabo India; ^4^ School of Food Science and Environmental Health Technological University Dublin – City Campus, Central Quad Dublin Ireland; ^5^ Centre for Sustainable Packaging and Bioproducts (CSPB) Technological University Dublin – City Campus, Central Quad Dublin Ireland

**Keywords:** Aonla, Giloy, process optimization, response surface methodology (RSM), spray drying

## Abstract

The combination of Giloy (*Tinospora cordifolia*) and Aonla ( *Phyllanthus emblica* L.) offers significant health benefits, but the high perishability of their juices limits stability and bioactive compound retention. Spray drying presents a promising solution to enhance shelf life while preserving key nutrients. This study optimized the spray drying process for Aonla–Giloy juice using response surface methodology (RSM), evaluating powder recovery, moisture content, ascorbic acid, total phenolic content, color change, hygroscopicity, dispersibility, water solubility index, and overall acceptability. The effects of feed flow rate (2.1–4.1 mL/min), inlet air temperature (150°C–190°C), Aonla juice percentage (30%–70%), and maltodextrin concentration (20%–30%) were investigated. Among these, maltodextrin concentration had the most significant impact, followed by inlet air temperature, Aonla juice percentage, and feed flow rate. The optimized conditions (2.7 mL/min feed flow, 167°C inlet air temperature, 70% Aonla juice, 21.5% maltodextrin) resulted in high powder recovery (20.31%), ascorbic acid (675.93 mg/100 g), phenolic content (23,397.43 mg GAE/100 g), dispersibility (89.23%), solubility index (90.24%), and overall acceptability (86.37%), while maintaining low moisture content (6.34%), hygroscopicity (38.26%), and minimal color change (ΔE = 15.99). This study demonstrates the practical potential of spray drying for developing shelf‐stable, bioactive‐rich powders from medicinal plant‐based juices, offering applications in the functional food and nutraceutical industries.

## Graphical abstract

This graphical abstract presents the preparation, optimization, and characterization of spray‐dried Aonla–Giloy juice powder. Fresh Aonla fruit and Giloy stem were processed through extraction, pre‐treatment, filtration, blending, maltodextrin addition, and spray drying, followed by RSM‐based optimization of key formulation and drying variables. The study evaluates the influence of processing conditions on powder recovery, moisture content, bioactive retention, color, hygroscopicity, dispersibility, solubility, and sensory acceptability. The developed powder demonstrated desirable physicochemical, functional, reconstitution, and sensory properties, supporting its potential as a shelf‐stable functional food and nutraceutical ingredient.

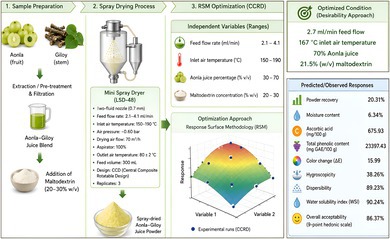

**Abbreviations**
AA: ascorbic acid
ANOVA: analysis of variance
AOAC: Association of Official Analytical Chemists
CCRD: central composite rotatable design
MC: moisture content
OA: overall acceptability
RSM: response surface methodology
RTS: ready‐to‐serve
TPC: total phenolic content
TSS: total soluble solids
WSI: water solubility index

## 1 Introduction

A notable shift in consumer preferences from highly processed, ready‐to‐eat foods to natural and minimally processed foods has emerged due to increasing awareness of healthy eating habits. Numerous studies have highlighted the significance of phytochemicals, particularly plant‐derived compounds, in the prevention and management of chronic diseases (Ginwala et al. 2019; Rupasinghe 2020). Ascorbic acid (vitamin C) plays a crucial role in various physiological functions, including collagen synthesis, immune defense, and antioxidant protection. Its ability to enhance collagen formation, support immune cells, and neutralize free radicals makes it essential for overall health. High doses of ascorbic acid (AA) have been linked to the prevention and treatment of cancer, diabetes, atherosclerosis, the common cold, cataracts, glaucoma, macular degeneration, stroke, and high cholesterol levels (Chambial et al. 2013). As an antioxidant, it shields the body from oxidative stress, toxins, and environmental pollutants. Similarly, studies on humans, animals, and epidemiological models indicate that polyphenols exhibit potent antioxidant and anti‐inflammatory properties, which may contribute to the prevention and management of obesity, cancer, neurodegenerative diseases, and cardiovascular disorders (Singh et al. 2011).

*Tinospora cordifolia*, commonly known as Giloy, is a widely used medicinal herb, particularly in pharmaceutical formulations. It contains alkaloids, sesquiterpenoids, aliphatic compounds, glycosides, steroids, essential oils, fatty acid derivatives, and polysaccharides, which contribute to its therapeutic properties, including its role in enhancing reproductive health and regulating blood and lipid profiles (Premkumar et al. 2021). Additionally, Giloy has been traditionally used to treat tuberculosis, gout, and jaundice, though scientific validation for some of these applications remains limited.

The plant is primarily found in India, Malaysia, and Sri Lanka, with only four of its 40 known species identified in India. The stem and root are the key medicinal parts, and the nutrient‐rich starch extracted from Giloy stems is widely used in herbal medicine. The extract has a mild bittersweet taste with no distinct flavor, making it ideal for blending with fruit juices to enhance consumer appeal.

Aonla ( *Phyllanthus emblica* L.), a highly nutritious fruit, is well‐suited for blending with Giloy due to its high vitamin C content (500–1500 mg/100 g), pectin (2.25%–11.19%), and polyphenols (24.61%–31.12%) (Devi et al. 2020). In India, Aonla is a key component in traditional medicine and is widely used in formulations such as Triphala and Chyawanprash. Despite its exceptional nutritional and medicinal value, Aonla remains underutilized in international markets, primarily due to limited consumer awareness. The fruit is highly perishable, acidic, and astringent, making it less preferred for direct consumption (Priya and Khatkar 2013). However, it is extensively processed into value‐added products such as preserves, candies, jellies, toffees, pickles, fruit leathers, squashes, juices, ready‐to‐serve (RTS) beverages, cider, dried powders, and various Ayurvedic formulations, including Chyawanprash, Triphala, Amrit Kalash, and Amol Ki Rasayan (Meena et al. 2022).

Aonla and Giloy are widely used in functional beverages due to their high antioxidant content and health‐promoting properties. However, these beverages have a limited shelf life due to natural biochemical changes that affect their stability and quality over time. Studies on blended RTS beverages containing aonla indicate that these products undergo notable changes in total soluble solids (TSS), pH, and acidity during storage. For example, an RTS beverage combining kinnow and aonla exhibited a significant increase in TSS over 120 days, likely due to polysaccharide breakdown into simpler sugars, while a decline in acidity impacted flavor and overall shelf stability (Das et al. 2021).

Similarly, research on antioxidant‐rich RTS beverages incorporating aonla with carrot and ginger has shown a progressive decline in total antioxidant activity during storage, further limiting shelf life (Pandey et al. 2020). Additionally, sensory attributes such as flavor, color, and overall acceptability (OA) deteriorate over time, affecting the commercial viability of these beverages under ambient storage conditions.

To maintain the nutritional and sensory properties of such products, advanced processing techniques are essential. These methods extend shelf life, enable year‐round availability, and ensure consistent product quality. Various techniques, including heat inactivation of enzymes (Yusof et al. 2000), freezing (Songsermpong and Jittanit 2010), blanching, or the use of antimicrobial and antioxidant agents (Ozoglu and Bayındırlı 2002), low‐temperature storage, and gamma radiation (Alcarde et al. 2003), have been employed to preserve fruit beverages. Among these, spray drying is emerging as a rapid, cost‐effective, and adaptable alternative to conventional drying techniques, allowing for the conversion of juices into stable, nutrient‐rich powders with extended shelf life (Tontul and Topuz 2017).

Spray drying is a widely used and highly effective method for preserving fruit juices, converting them into stable, powdered forms while retaining their nutritional and sensory properties. This process involves atomizing the feed solution into fine droplets using either a spray disc or a two‐fluid nozzle atomizer, followed by rapid water evaporation under controlled conditions. A key advantage of spray drying is the incorporation of carrier agents, which protect bioactive compounds from oxidation and degradation, thereby enhancing product stability (Chranioti et al. 2015; Schuck et al. 2016).

This method offers several benefits, including extended shelf life by reducing moisture content (MC), which helps prevent microbial growth and spoilage. Additionally, flavor, color, and nutritional value are well preserved, making spray‐dried powders highly desirable for food and nutraceutical applications. The resulting lightweight, easy‐to‐handle powders provide cost‐effective storage and transport solutions, making them particularly valuable for food manufacturers (Benavides‐Morán et al. 2021). However, spray drying of fruit juices is often associated with challenges such as wall deposition and stickiness, primarily due to the high concentration of organic acids and low‐molecular‐weight sugars present in the juice. These components lower the glass transition temperature of the solids, leading to adhesion of particles to the dryer wall and reduced powder recovery. To mitigate these issues and improve drying efficiency, carrier agents (high‐molecular‐weight additives) are commonly incorporated during the spray drying of fruit juices (Qadri et al. 2023). Besides the concentration of carrier agents, several process parameters such as inlet air temperature, feed solid concentration, and feed rate also significantly influence the physicochemical properties of spray‐dried powders. Inlet air temperature affects the drying rate and moisture removal, thereby influencing powder MC, particle morphology, and dispersibility. Feed solid concentration determines the viscosity and total solids of the feed, which affects droplet formation, particle size, and powder yield. Similarly, feed rate influences the residence time of droplets in the drying chamber; higher feed rates may reduce drying efficiency and increase powder MC. Therefore, these operating parameters play a critical role in determining the quality attributes of spray‐dried powders, including MC, bulk density, hygroscopicity, and solubility (Cruz‐Padilla et al. 2023; Ozdikicierler et al. 2014; Nadali et al. 2022; Santhalakshmy et al. 2015; Vargas et al. 2024).

Despite the well‐documented therapeutic properties of Aonla and Giloy, the development of stable powder products from their combined juices remains challenging due to the high perishability of the juice and the degradation of sensitive bioactive compounds during processing. Given these advantages, spray drying is an essential unit operation in the food industry, enabling the conversion of perishable fruit juices into stable, nutrient‐rich powders. The key question addressed in this study is how spray‐drying parameters and formulation variables can be optimized to produce a stable Aonla–Giloy juice powder with high bioactive retention and desirable physicochemical properties. The strength of this work lies in the systematic optimization of both process parameters and formulation composition using response surface methodology (RSM), providing a comprehensive understanding of their combined effects on powder recovery, functional quality attributes, and bioactive compound retention. Based on this background, the present study aimed to evaluate the impact of feed flow rate, inlet air temperature, Aonla juice percentage in the blend, and maltodextrin concentration on the physicochemical, powder, and reconstitution properties, as well as the sensory quality of spray‐dried Aonla–Giloy juice powder.

## 2 Materials and Methods

### 2.1 Materials

Freshly harvested Aonla fruits and Giloy stems were procured from the farms of Punjab Agricultural University, Ludhiana, India. The fruits were manually sorted to remove bruised, damaged, or physically injured samples. The selected fruits and stems were then thoroughly washed with water to eliminate field dirt, dust, and other impurities, ensuring cleanliness before further processing. All chemicals and reagents (Maltodextrin, and Folin–Ciocalteu reagent) used for the determination of AA, titratable acidity, and phenolic acids were of analytical reagent grade and procured from reputed suppliers such as Sigma‐Aldrich (Ludhaian, Punjab, India).

### 2.2 Sample Preparation

The extraction of Aonla and Giloy juice was carried out under optimized conditions determined through preliminary trials. For Aonla juice extraction, a cold extraction method was used. The fruits were deseeded, crushed using a pulping machine, and filtered through muslin cloth to obtain the juice. For Giloy juice extraction, the stems were chopped into small pieces and pretreated with 1% citric acid (w/v) solution in a water bath (HV‐135‐WB, India) at 40°C for 1 min, maintaining a fruit‐to‐solution ratio of 1:10. The juice was then further processed by cooking the Giloy stem mixture in an autoclave at 120°C and 15 psi for 2 h, using a giloy‐to‐water ratio of 1:4 to soften the fibrous stem matrix and facilitate the extraction of bioactive compounds prior to further processing. Both extracted and preprocessed juices (Aonla and Giloy) were clarified by filtering through cheesecloth to remove larger particles, ensuring a smooth feed solution and preventing clogging of the spray dryer atomizer nozzle.

Prior to spray drying, the feed solutions were characterized for TSS (°Brix), viscosity, and total solids content. TSS were measured using a digital refractometer and were found to be 8°–9° Brix for Aonla juice and 0.80°–1.17° Brix for Giloy juice. The viscosity of the feed solution was determined using a Brookfield viscometer and recorded as 9.42 cP for Aonla juice and 1.04 cP for Giloy juice. Total solids were measured by oven drying at 105°C to constant weight, yielding 11.97% for Aonla juice and 3.77% for Giloy juice.

### 2.3 Spray Drying

**FIGURE 1:**
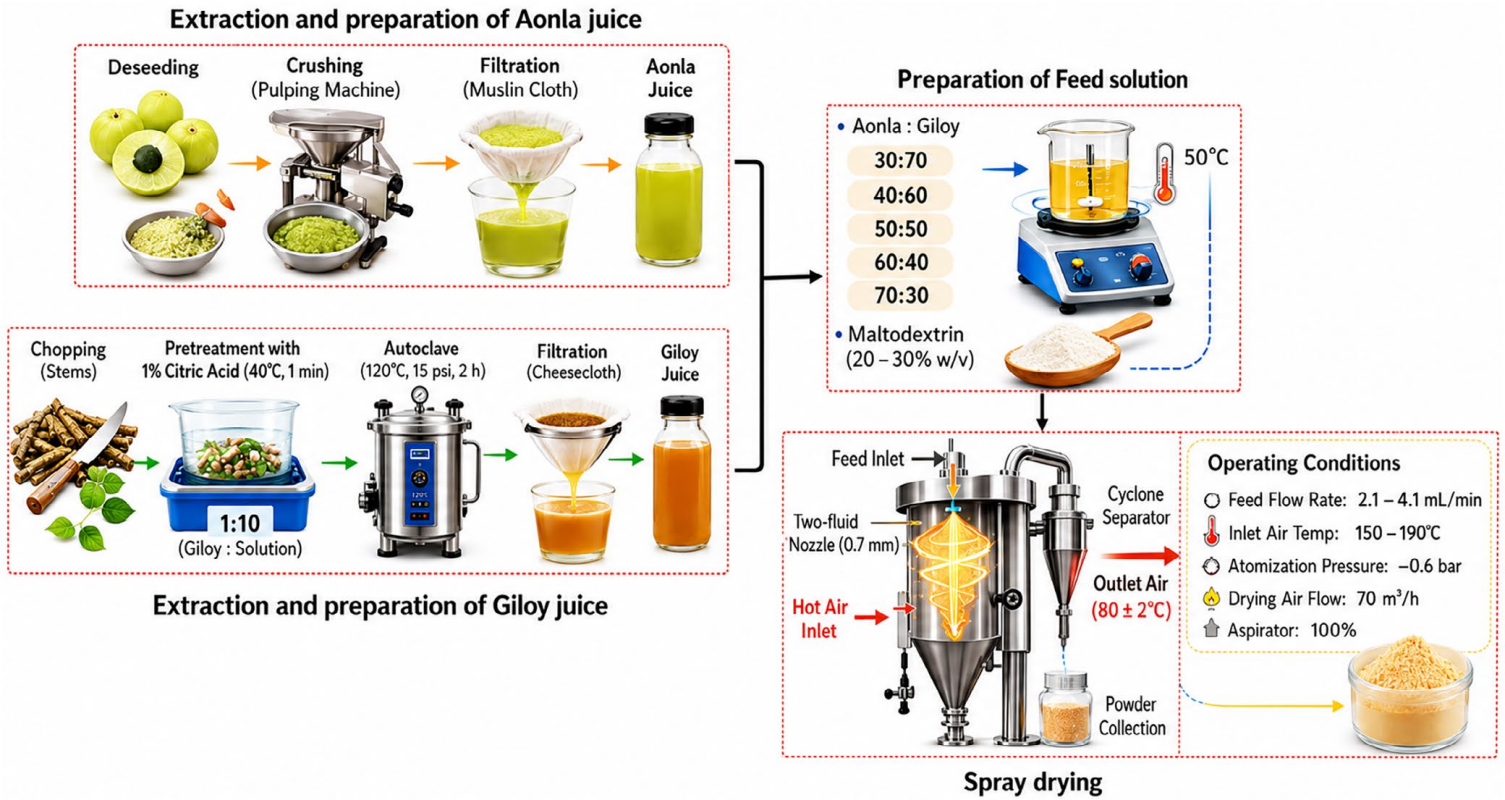
Schematic illustration for the preparation of spray‐dried Aonla–Giloy juice powder.

Aonla and Giloy juices were blended in different proportions, with the Aonla:Giloy juice percentage varying as 30%:70%, 40%:60%, 50%:50%, 60%:40% and 70%:30% (v/v). Maltodextrin (20.00%–30.00% w/v of the initial juice) was added as a carrier agent, and the mixture was stirred at 500 rpm using a magnetic stirrer while being heated to 50°C until completely dissolved. The prepared juice blend was then fed into a mini spray dryer (Model: LSD‐48, JISL Pvt. Ltd., Mumbai, India) equipped with a two‐fluid pneumatic atomizing nozzle (0.7 mm diameter). The feed was introduced at varying feed flow rates (2.10–4.10 mL/min) while maintaining inlet air temperatures in the range of 150°C–190°C. The atomization air pressure was maintained at approximately 0.60 bar, while the drying air flow rate and aspirator were maintained at 70.00 m^3^/h and 100% capacity, respectively. During the drying process, the outlet air temperature ranged between 80°C ± 2°C, depending on the operating conditions, and was continuously monitored to evaluate drying performance. The resultant spray‐dried powders were immediately collected from the cyclone separator, weighed to determine powder yield, and stored in sealed glass jars for further analysis. The central composite rotatable design (CCRD) was used to determine the experimental runs, with the total juice volume for each spray drying trial set at 300.00 mL. All experiments were conducted in triplicate to ensure data reliability (Table 1). A schematic illustration of complete process for the preparation of spray‐dried Aonla–Giloy juice powder is given in Figure 1.

**TABLE 1:** Experimental design matrix for spray drying of Aonla–Giloy juice using a four‐factor central composite rotatable design.

Run	Feed flow rate (mL/min)	Inlet air temperature (°C)	Aonla juice (%) in blended juice	Maltodextrin concentration (%)
1	3.10	170	50.00	30.00
2	3.60	180	60.00	27.50
3	3.60	160	60.00	22.50
4	2.60	180	40.00	27.50
5	2.10	170	50.00	25.00
6	4.10	170	50.00	25.00
7	3.60	180	60.00	22.50
8	3.60	160	60.00	27.50
9	3.10	190	50.00	25.00
10	3.60	160	40.00	22.50
11	3.10	170	50.00	25.00
12	2.60	160	60.00	27.50
13	3.10	170	50.00	20.00
14	3.10	170	70.00	25.00
15	3.10	170	50.00	25.00
16	3.60	180	40.00	27.50
17	3.60	160	40.00	27.50
18	2.60	160	40.00	22.50
19	2.60	160	40.00	27.50
20	3.10	170	30.00	25.00
21	2.60	160	60.00	22.50
22	3.60	180	40.00	22.50
23	2.60	180	60.00	27.50
24	3.10	170	50.00	25.00
25	2.60	180	40.00	22.50
26	2.60	180	60.00	22.50
27	3.10	150	50.00	25.00

### 2.4 Characterization of Spray‐Dried Aonla–Giloy Mix Powder

The spray‐dried Aonla–Giloy mix powders were analyzed for powder recovery, hygroscopicity, MC, AA, and total phenolic content (TPC). Additionally, the reconstituted powders were evaluated for dispersibility, water solubility index (WSI), color, and sensory attributes to assess their functional and consumer acceptability.

#### 2.4.1 Physicochemical Properties

The MC (% wb) and titratable acidity (%) of the spray‐dried powder were determined using standard methods (Association of Official Analytical Chemists [AOAC] 2000). AA concentration was measured using the titration method (Ranganna 1986) and expressed as mg/100 g. The TPC was analyzed using the Folin–Ciocalteu reagent method (McDonald et al. 2001). Absorbance was measured at 765 nm using a UV–Vis spectrophotometer, with results expressed as mgGAE per 100 g, based on a gallic acid standard curve.

#### 2.4.2 Powder Recovery

Powder recovery was calculated based on the total solids content of the feed formulation. It was defined as the ratio of the mass of powder collected after spray drying to the total mass of solids present in the feed solution used during the process. Powder recovery was calculated using the following equation:

$$\text{Powder recovery}\;\left(\%\right)=\frac{\text{Weight of powder collected}\;\left(g\right)\;x\;100}{\text{Total solids in feed}\;\left(g\right)}$$

#### 2.4.3 Hygroscopicity

The hygroscopicity of the spray‐dried powder was determined following the method described by Cai and Corke (2000), with slight modifications. Approximately 3 g of powder was placed in a humidity chamber at 25°C and 81% relative humidity and weighed after 1 week. Hygroscopicity was expressed as the grams of adsorbed moisture per 100 g of powder, calculated using the equation:

$$\text{Hygroscopicity}\;\left(\%\right)=\frac{\text{total weight of water adsorbed during}\;\mathrm{one}\;\text{week}\;x\;100}{\text{weight of dried sample used for study}\;\left(g\right)}$$

#### 2.4.4 Dispersibility

The dispersibility of the reconstituted powder was assessed in accordance with the methodology outlined by (Frascareli et al. 2012), and the calculation was performed using the standardized equation provided below:

$$\text{Dispersibility}\;\left(\%\right)=\frac{\left(10+a\right)\times \%\mathrm{TS}}{a\times \left(100-b\right)/100}$$

where *a* = amount of powder being used (g), *b* = moisture content in powder (%), and %TS = percentage dry matter in the reconstituted juice after it has been passed through the sieve.

#### 2.4.5 Water Solubility Index

The WSI was measured following the method outlined by Jafari et al. (2017). A 2.5 g sample of spray‐dried Aonla–Giloy mix powder was suspended in 30 mL of distilled water and agitated in a vortex mixer for 1 min. The suspension was maintained at 37°C for 30 min and centrifuged at 3500 rpm at 4°C for 20 min. After centrifugation, an aliquot of the supernatant (equivalent to 0.2 g) was transferred to a pre‐weighed evaporating dish and dried at 105°C ± 1°C until constant weight. The WSI was calculated using the equation:

$$\mathrm{WSI}\;\left(\%\right)=\frac{S_{3}-S_{2}}{S_{1}}\times 100$$

where *S*_1_ = weight of the supernatant aliquot used for drying (0.2 g), *S*_2_ = weight of evaporating dish with dried product, and *S*_3_ = weight of evaporating dish with supernatant.

#### 2.4.6 Color

A Konica‐Minolta Chroma meter CR‐410 (Konica‐Minolta Sensing, Tokyo, Japan) was used to determine the color of samples on the basis of tristimulus parameters, that is, *L* (whiteness/darkness), *a* (redness/greenness) and *b* (yellowness/blueness) values (Muzaffar et al. 2016). The total color change of the fresh feed blend and spray‐dried Aonla–Giloy mix reconstituted powder was also determined as described by the following equation:

$$\text{Total color change},△E=\sqrt{{\left(L_{0}-L\right)}^{2}+{\left(a_{0}-a\right)}^{2}+{\left(b_{0}-b\right)}^{2}}$$

*L*, *a* and *b* denote the brightness, redness and yellowness of the juice and spray‐dried samples, respectively, where the subscript “0” is related to the color values of the corresponding fresh feed blend used as the control for that experimental run.

#### 2.4.7 Sensory Evaluation

Reconstituted juice was prepared by dissolving spray‐dried powder in distilled water at a 1:5 (w/v) ratio, following standard practice used for spray‐dried citrus beverage powders and based on preliminary trials to obtain a beverage‐like consistency suitable for sensory evaluation. Sensory analysis was conducted under controlled laboratory conditions at 25°C ± 2°C with uniform lighting. A panel of 20 semi‐trained members was selected based on their willingness to participate, absence of known allergies to the ingredients used in the formulation, and their ability to perceive and evaluate basic sensory attributes. The samples were randomly coded and presented to a panel of 20 semi‐trained members who were briefed on the evaluation attributes prior to testing. Participation of panelists was voluntary. The samples were evaluated using a 9‐point hedonic scale for appearance, aroma, taste, and OA.

### 2.5 Statistical Analysis and Optimization

Process conditions were optimized by CCRD using Design‐Expert software (version. 13.0.5, Stat‐Ease). A total of 27 experimental runs were obtained. The independent variables and their coded levels are given in Table 1 where feed flow rate, inlet air temperature, aonla to juice ratio and concentration of maltodextrin are denoted by (*X*_1_), (*X*_2_), (*X*_3_) and (*X*_4_) respectively. The experimental data were analyzed using analysis of variance (ANOVA) to evaluate the significance of model terms and their interactions. Statistical significance was determined using *F*‐values and corresponding *p*‐values (*p* < 0.05). Model adequacy was assessed using lack‐of‐fit test, where a nonsignificant lack‐of‐fit indicates good model fit.

## 3 Results and Discussion

### 3.1 Optimization of Spray Drying by CCRD

This study investigated the impact of spray drying on the physicochemical, functional, and reconstituted properties, as well as the sensory quality of the encapsulated powder. The study employed second‐order regression models to match data for all response variables, evaluating goodness of fit based on significance (*p* < 0.05) and lack of fit (*p* > 0.05). The results showed that all constructed models had significant *p*‐values (*p* < 0.05), while the lack of fit remained nonsignificant (*p* > 0.05), indicating a good model fit (Table 2).

**TABLE 2:** Analysis of variance (ANOVA) results for the regression analysis of spray drying responses of Aonla–Giloy mix juice.

Parameters Effects	df	*F*								
		Recovery	Moisture content	Ascorbic acid	Total phenolic content	Hygroscopicity	Dispersibility	Water solubility index	Total color change	Overall acceptability
Model	14	272.04 *	239.16*	358.24*	52.76*	580.82*	11.53*	336.32*	32.86*	155.52*
A‐Feed flow rate	1	287.17 *	131.87*	480.51*	11.35	680.72*	13.46*	1.64	5.55	906.18*
B‐Inlet air temperature	1	675.00*	1993.09*	873.72*	38.40*	3980.30*	55.65*	1305.01*	102.98*	369.15*
C‐Aonla juice (%) in blend	1	515.81*	89.29*	2533.08*	152.53*	74.11*	18.80*	669.68*	58.61*	211.88*
D‐Maltodextrin concentration	1	2247.34*	1088.40*	1093.49*	493.3*	3347.41*	60.38*	2708.69*	285.32*	675.73*
AB	1	0.40	21.37*	1.44	0.76	2.37	1.15	0.001	0.007	0.39
AC	1	0.31	0.0435	3.90	0.0059	0.045	0.0046	0.0004	0.004	0.24
AD	1	1.31	0.5608	1.93	0.02	2.03	0.014	0.0002	0.018	0.66
BC	1	0.72	0.546	7.38*	0.032	0.26	0.012	0.35	0.076	0.094
BD	1	3.00	7.034*	3.66	0.11	11.50*	0.036	1.41	0.34	0.25
CD	1	2.36	0.3018	9.92*	0.32	0.22	0.012	0.74	0.003	0.16
A^2^	1	0.00999	0.2208	0.14	0.94	0.015	0.893	1.13	0.66	0.012
B^2^	1	2.58	3.63	4.22	39.82*	18.93*	10.75*	5.27*	0.22	8.26*
C^2^	1	0.088	0.068	0.12	1.27	0.047	1.708	1.71	0.68	0.29
D^2^	1	60.93*	9.554*	6.12E‐04	2.85	13.7*	4.058	6.91*	6.93*	0.28
Lack of fit	10	0.20	0.21	0.56	11.55	0.11	4.54	0.37	0.13	0.45
*R* ^2^ (Adjustd *R* ^2^)		0.996 (0.993)	0.996 (0.992)	0.998 (0.995)	0.984 (0.965)	0.998 (0.997)	0.931 (0.850)	0.997 (0.994)	0.974 (0.949)	0.982 (0.961)

* Indicates that the *p* value is significant at the 5% level.

The regression equations, utilized to establish the relationship between the response values and test variables (feed flow rate, inlet air temperature, percentage of Aonla juice in blended juice, and maltodextrin concentration), are presented in Table 3, while the experimental data from 27 runs are shown in Tables 4 and 5. A good‐fit model should have a coefficient of determination (*R* ^2^) of at least 80% (> 0.80), which indicates how much variance in the response can be attributed to the model rather than random error (Kaur et al. 2024). The study of the model's results revealed that all response variable models were adequate, with satisfactory *R* ^2^ values (> 0.90). Additionally, the computed adjusted *R* ^2^ and predicted *R* ^2^ values differed by < 20.00%, further confirming model reliability.

**TABLE 3:** Regression models developed for predicting the effects of process variables on spray‐dried Aonla–Giloy mix powder properties.

Regression equation	Best model	*R* ^2^	Adjusted *R* ^2^	CV (%)
Moisture content = +5.83 + 0.14 × A−0.56 × B−0.12 × C−0.42 × D−0.07 × A × B−0.0032 × A × C−0.012 × A × D + 0.011 × B × C + 0.04 × B × D + 0.00848 × C × D + 0.00628 × A^2^−0.025 × B^2^ + 0.00348 × C^2^−0.04 × D^2^	Quadratic	0.996	0.992	1.07
Ascorbic acid = +520.48 + 25.33 × A−34.16 × B + 58.16 × C−38.21 × D−1.70 × A × B + 2.79 × A × C−1.97 × A × D−3.85 × B × C + 2.71 × B × D−4.46 × C × D + 0.46 × A^2^−2.52 × B^2^ + 0.42 × C^2^ + 0.030 × D^2^	Quadratic	0.997	0.994	1.09
Total phenolic content = +20,306.08 + 172.31 × A−316.98 × B + 631.72 × C−1136.07 × D−54.76 × A × B + 4.79 × A × C−8.79 × A × D−11.22 × B × C + 20.57 × B × D−35.64 × C × D + 52.67 × A^2^ + 342.36 × B^2^ + 61.08 × C^2^ + 91.58 × D^2^	Quadratic	0.984	0.965	1.21
Powder recovery = +21.64–0.65 × A−1.00 × B + 0.87 × C + 1.82 × D + 0.030 × A × B−0.026 × A × C−0.054 × A × D−0.040 × B × C−0.082 × B × D + 0.072 × C × D + 0.004075 × A^2^−0.065 × B^2^ + 0.012 × C^2^−0.32 × D^2^	Quadratic	0.996	0.993	0.88
Hygroscopicity = +35.77−1.08 × A + 2.62 × B + 0.36 × C−2.40 × D−0.078 × A × B−0.011 × A × C + 0.072 × A × D + 0.026 × B × C−0.17 × B × D−0.024 × C × D + 0.0053 × A^2^−0.19 × B^2^−0.0095 × C^2^−0.16 × D^2^	Quadratic	0.998	0.996	0.57
Dispersibility = +87.20−1.18 × A + 2.40 × B + 1.40 × C−2.50 × D + 0.42 × A × B−0.027 × A × C + 0.047 × A × D + 0.043 × B × C−0.075 × B × D−0.043 × C × D−0.32 × A^2^−1.12 × B^2^−0.45 × C^2^−0.69 × D^2^	Quadratic	0.931	0.850	1.86
Water solubility index = +72.15−1.02 × A + 3.59 × B + 1.46 × C−5.06 × D−0.050 × A × B−0.021 × A × C + 0.070 × A × D + 0.072 × B × C−0.25 × B × D−0.10 × C × D + 0.032 × A^2^–0.17 × B^2^ + 0.040 × C^2^−0.047 × D^2^	Quadratic	0.997	0.994	0.38
Total color change (ΔE) = +15.37 + 0.16 × A−0.67 × B + 0.51 × C + 1.12 × D−0.0067 × A × B + 0.0052 × A × C + 0.011 × A × D−0.022 × B × C−0.047 × B × D−0.005 × C × D + 0.057 × A^2^ + 0.033 × B^2^ + 0.058 × C^2^ + 0.18 × D^2^	Quadratic	0.974	0.944	2.07
Overall acceptability = +78.01−3.18 × A + 2.03 × B + 1.54 × C−2.74 × D−0.080 × A × B−0.064 × A × C + 0.10 × A × D + 0.040 × B × C−0.065 × B × D−0.052 × C × D + 0.012 × A^2^−0.32 × B^2^ + 0.061 × C^2^ + 0.059 × D^2^	Quadratic	0.994	0.988	0.66

**TABLE 4:** Effect of feed flow rate, inlet air temperature, Aonla juice percentage (%) in blended juice and maltodextrin concentration on the physicochemical and powder properties of Aonla–Giloy mix powder.

Feed flow rate (mL/min)	Inlet air temperature (°C)	Aonla juice (%) in blended juice	Maltodextrin concentration (%)	Moisture Content (%)	Ascorbic acid (mg/100 g)	Total phenolic content (mg GAE/100 g)	Recovery (%)	Hygroscipicity (g/100 g)
3.10	170	50.00	30.00	4.82	453.37	18,325.30	24.29	30.27
3.60	180	60.00	27.50	4.83	519.55	20,133.73	22.15	34.68
3.60	160	60.00	22.50	6.92	694.61	23,436.38	20.54	34.30
2.60	180	40.00	27.50	4.86	377.47	18,755.80	21.69	36.12
2.10	170	50.00	25.00	5.60	472.09	19,899.86	23.05	38.02
4.10	170	50.00	25.00	6.06	573.83	20,707.88	20.41	33.66
3.60	180	60.00	22.50	5.58	607.23	22,515.55	18.74	39.67
3.60	160	60.00	27.50	5.98	594.31	20,957.15	24.28	29.99
3.10	190	50.00	25.00	4.58	445.33	21,034.38	19.37	40.48
3.60	160	40.00	22.50	7.21	556.39	22,051.29	18.93	33.62
3.10	170	50.00	25.00	5.73	513.38	20,299.85	21.69	35.37
2.60	160	60.00	27.50	5.57	539.06	20,544.21	25.81	31.87
3.10	170	50.00	20.00	6.45	589.12	22,593.73	16.59	40.06
3.10	170	70.00	25.00	5.57	642.57	21,574.93	23.53	36.50
3.10	170	50.00	25.00	5.82	527.59	20,299.85	21.29	36.17
3.60	180	40.00	27.50	5.03	416.16	18,943.83	20.41	33.99
3.60	160	40.00	27.50	6.23	476.04	19,718.58	22.38	29.39
2.60	160	40.00	22.50	6.70	504.66	21,616.79	20.12	35.73
2.60	160	40.00	27.50	5.80	431.79	19,330.04	23.79	31.24
3.10	170	30.00	25.00	6.06	403.01	19,100.13	19.99	35.06
2.60	160	60.00	22.50	6.44	630.03	22,974.59	21.83	36.46
3.60	180	40.00	22.50	5.81	486.39	21,184.88	17.27	38.88
2.60	180	60.00	27.50	4.67	471.24	19,933.89	23.54	36.85
3.10	170	50.00	25.00	5.94	520.48	20,299.85	21.94	35.77
2.60	180	40.00	22.50	5.62	441.17	20,974.61	18.35	41.32
2.60	180	60.00	22.50	5.40	550.78	22,292.07	19.91	42.16
3.10	150	50.00	25.00	6.82	576.78	21,890.88	23.53	29.62

**TABLE 5:** Effect of feed flow rate, inlet air temperature, Aonla juice concentration (%) in blended juice and maltodextrin concentration on the reconstitutional properties of the Aonla–Giloy mix powder.

Feed flow rate (mL/min)	Inlet air temperature (°C)	Aonla juice (%) in blended juice	Maltodextrin concentration (%)	Dispersibility (%)	Water solubility index (%)	Total color change (ΔE)	Overall acceptability (%)
3.10	170	50.00	30.00	80.81	74.63	18.19	72.26
3.60	180	60.00	27.50	84.18	82.57	16.84	75.39
3.60	160	60.00	22.50	83.25	84.74	16.03	76.56
2.60	180	40.00	27.50	83.50	79.46	15.53	78.58
2.10	170	50.00	25.00	88.11	82.43	15.09	84.64
4.10	170	50.00	25.00	86.37	82.10	15.70	71.89
3.60	180	60.00	22.50	89.38	89.60	14.71	80.54
3.60	160	60.00	27.50	78.41	78.09	18.35	71.66
3.10	190	50.00	25.00	88.47	86.10	13.98	81.22
3.60	160	40.00	22.50	80.45	81.38	14.93	73.54
3.10	170	50.00	25.00	86.38	82.27	14.74	78.01
2.60	160	60.00	27.50	82.32	78.25	17.99	77.76
3.10	170	50.00	20.00	90.74	87.89	13.63	84.64
3.10	170	70.00	25.00	89.37	85.66	16.36	81.43
3.10	170	50.00	25.00	87.97	82.66	15.98	78.71
3.60	180	40.00	27.50	81.34	79.30	15.85	72.42
3.60	160	40.00	27.50	75.77	75.00	17.27	68.84
2.60	160	40.00	22.50	84.45	81.54	14.63	79.80
2.60	160	40.00	27.50	79.54	75.15	16.93	74.70
3.10	170	30.00	25.00	84.12	79.01	14.44	75.49
2.60	160	60.00	22.50	87.40	84.91	15.71	83.07
3.60	180	40.00	22.50	86.37	86.05	13.70	77.37
2.60	180	60.00	27.50	86.41	82.74	16.51	81.80
3.10	170	50.00	25.00	87.24	81.75	15.40	77.30
2.60	180	40.00	22.50	88.66	86.22	13.43	83.95
2.60	180	60.00	22.50	91.75	89.78	14.42	87.39
3.10	150	50.00	25.00	79.63	76.61	16.62	72.64

**FIGURE 2:**
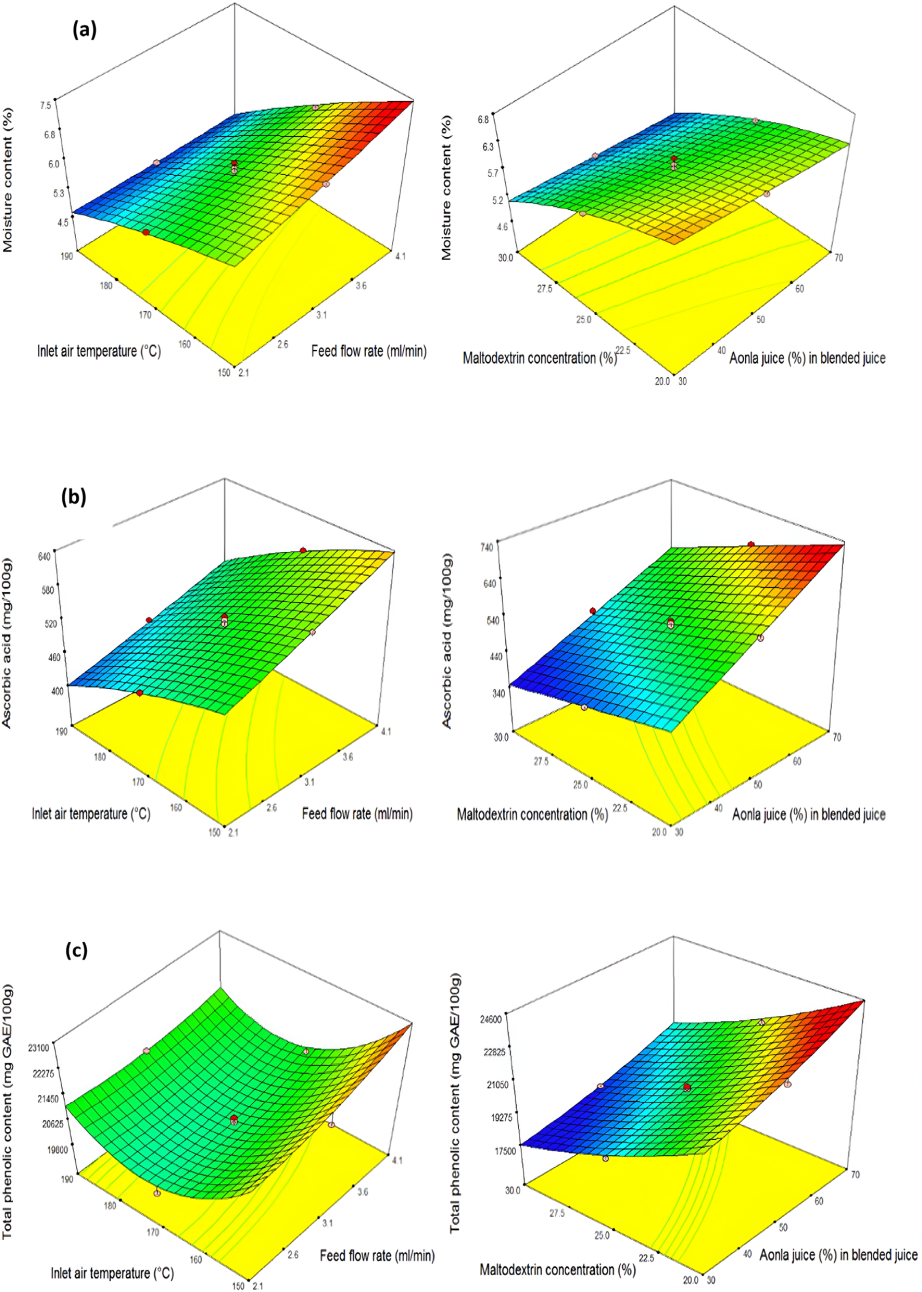
(a) Moisture content, (b) ascorbic acid, and (c) total phenolic content of spray‐dried Aonla–Giloy mix powder as influenced by different process conditions.

**FIGURE 3:**
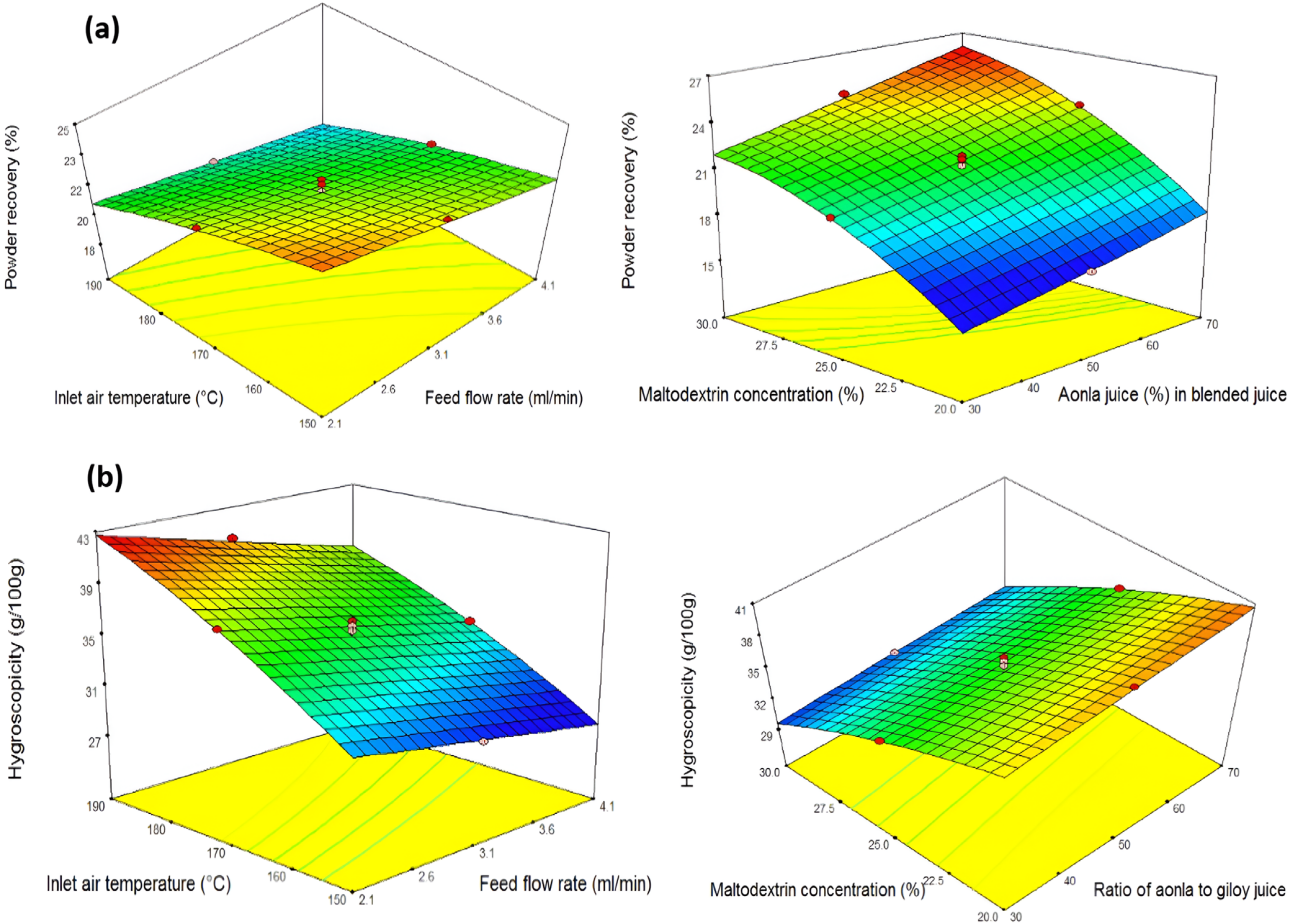
(a) Powder recovery and (b) hygroscopicity of spray‐dried Aonla–Giloy mix powder under various drying parameters.

**FIGURE 4:**
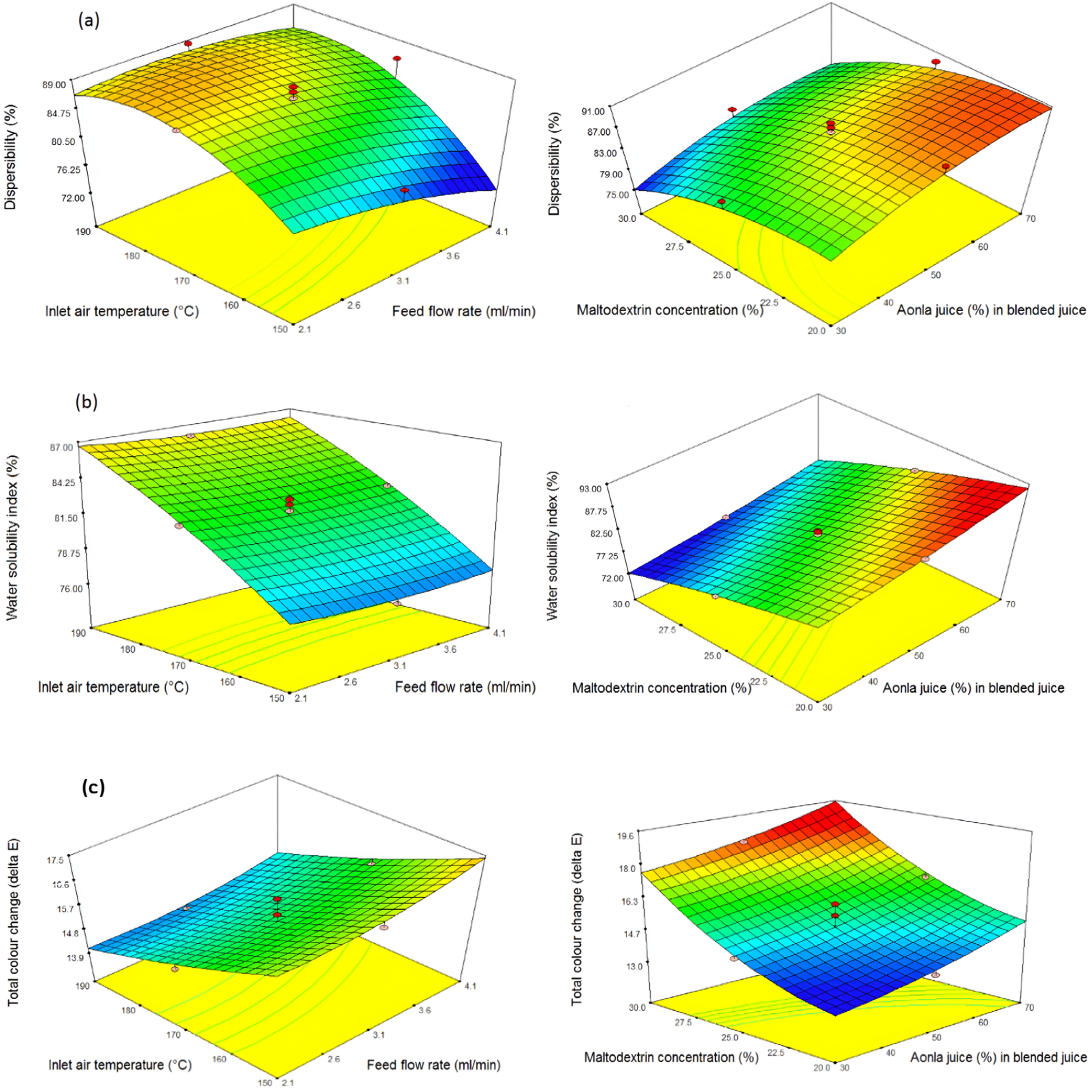
(a) Dispersibility, (b) water solubility index (WSI), and (c) total color change (ΔE) of spray‐dried Aonla–Giloy mix powder as affected by spray‐drying conditions.

**FIGURE 5:**
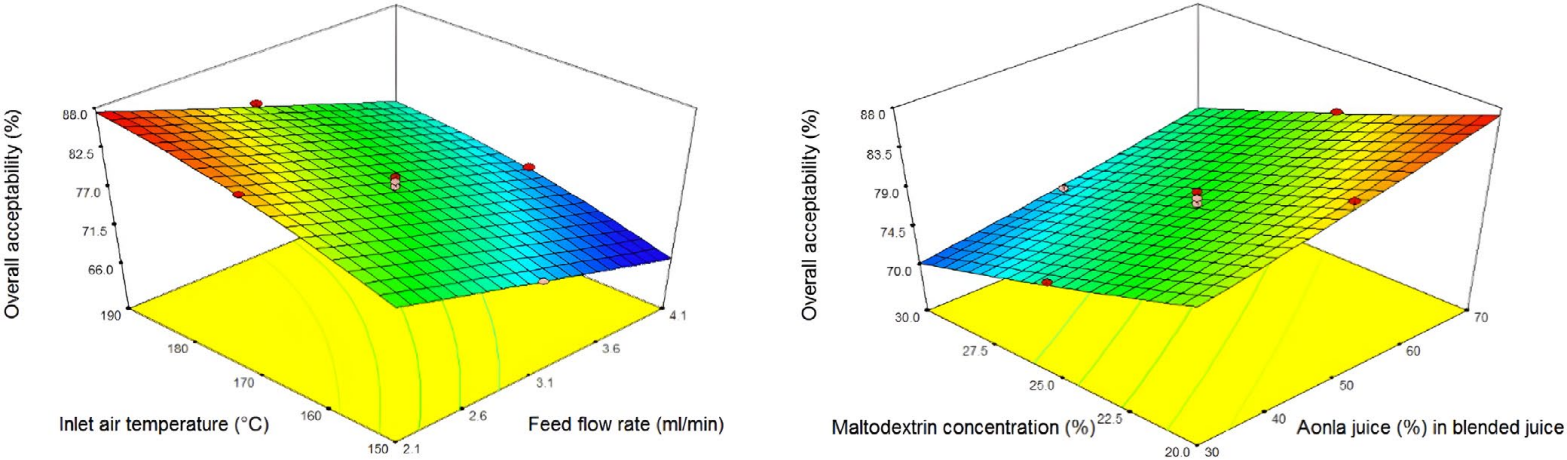
Overall acceptability of spray‐dried Aonla–Giloy mix powder evaluated under different formulation and processing settings.

Tables 4 and 5 revealed varying MC (4.58% to 7.21%), AA content (377.47 to 694.61 mg/100 g), and TPC (18,325.30 to 23,436.38 mg GAE/100 g). Powder properties, including recovery (16.59% to 25.81%) and hygroscopicity (29.39 to 42.16 g/100 g), were assessed. The dispersibility, WSI, and total color change exhibited ranges of 75.77% to 91.75%, 74.63% to 89.78%, and 13.43 to 18.35, respectively. Response surface plots for each response were generated for the different interactions of two independent variables and are presented in Figures 2, 3, 4, 5. The effects of spray drying on the physicochemical quality, powder and reconstitution properties, and sensory evaluation of the dried powder are discussed in detail in the following sections.

### 3.2 Effects of Spray Drying on the Quality Characteristics of Blended Juice Powder

#### 3.2.1 Moisture Content

The MC of a powder is a critical quality parameter affecting its flowability, stickiness, and storage stability, primarily due to variations in glass transition temperature and crystallization behavior during drying (Shrestha et al. 2007). The variation in MC of the blended juice powder, as influenced by spray drying parameters, is presented in Table 4. The MC of the dried powder ranged from 4.58% to 7.21% and was significantly affected by feed flow rate, inlet air temperature, Aonla juice percentage in the blend, and maltodextrin concentration (Table 2). The interaction effects of feed flow rate‐inlet air temperature and inlet air temperature‐maltodextrin concentration, along with the quadratic effect of maltodextrin concentration, had a significant (*p* < 0.05) impact on MC. Among all process variables, the linear effect of inlet air temperature exhibited the highest *F*‐value, indicating its dominant role in determining the MC of the Aonla–Giloy powder.

As depicted in Figure 2a, MC decreased with increasing inlet air temperature, Aonla juice percentage, and maltodextrin concentration. The reduction in MC at higher inlet air temperatures may be attributed to the rapid evaporation of moisture due to high‐temperature exposure, which enhances drying efficiency (Kha et al. 2010). Additionally, the decrease in MC with increasing Aonla juice percentage could be attributed to the higher TSS of Aonla juice compared to Giloy juice. A higher TSS results in less free water availability in the feed, leading to lower MC in the final powder. Similarly, an increase in maltodextrin concentration contributed to higher feed solid content, which in turn reduced the overall moisture available for evaporation. These findings align with those reported by Mishra et al. (2014), Muzaffar et al. (2016), and Vivek et al. (2021). Conversely, MC increased with increasing feed flow rate, likely due to shortened exposure time of feed droplets to hot air, resulting in incomplete moisture evaporation. A similar trend has been observed in previous studies (Patil et al. 2014).

#### 3.2.2 Ascorbic Acid

Ascorbic acid, commonly known as vitamin C (C_6_H_8_O_6_), is a naturally occurring antioxidant widely used as a functional food additive. The variation in AA content of the blended juice powder due to spray drying is presented in Table 4 and Figure 2b. The AA content of the dried powder ranged from 377.47 to 694.61 mg/100 g and was significantly (*p* < 0.05) influenced by the linear effects of feed flow rate, inlet air temperature, Aonla juice percentage in the blend, and maltodextrin concentration, as indicated by ANOVA results (Table 2). Additionally, interaction effects between inlet air temperature and Aonla juice percentage and between Aonla juice percentage and maltodextrin concentration also had a significant (*p* < 0.05) impact on AA retention. Among all process variables, the Aonla juice percentage in the blended juice had the most dominant effect on AA content, as evidenced by the highest *F*‐value.

As shown in Figure 2b, the AA content of the powder increased with both feed flow rate and Aonla juice percentage in the blend. This increase in AA with higher feed flow rate may be attributed to the shorter residence time of the droplets in the drying chamber, which reduces heat exposure and minimizes thermal degradation (Dias et al. 2018). Furthermore, the higher AA content in powders with increased Aonla juice percentage is expected, given the naturally high vitamin C concentration in Aonla juice.

Conversely, AA concentration was negatively affected by inlet air temperature and maltodextrin concentration. Elevated drying temperatures likely accelerated the thermal degradation of AA, leading to a reduction in AA retention. As temperatures rise, the conversion of AA into 2,3‐diketogulonic acid accelerates, causing a significant decline in vitamin C activity (Moreau et al. 2024; Villota and Hawkes 2018). A similar trend was reported by Santhalakshmy et al. (2015) for spray‐dried jamun fruit juice powder and by Patil et al. (2014) in the manufacturing of guava powder. The decline in AA content with increasing maltodextrin concentration can be attributed to the dilution effect, where a higher proportion of carrier agent results in a lower concentration of bioactive compounds in the final powder (Mishra et al. 2014).

#### 3.2.3 Total Phenolic Content

TPC is an important plant constituent with redox properties, which contribute significantly to antioxidant activity. The variation in TPC of the Aonla–Giloy mix powder in response to spray drying parameters is presented in Table 4 and Figure 2c. The TPC of the spray‐dried powder ranged from 18,325.30 to 23,436.38 mg GAE/100 g. ANOVA results indicated that the linear effects of feed flow rate, inlet air temperature, Aonla juice percentage in the blend, and maltodextrin concentration, as well as the quadratic effect of inlet air temperature, had significant (*p* < 0.05) influences on the TPC of the powder (Table 2).

An increase in maltodextrin concentration had a dominant but negative effect on TPC, likely due to a dilution effect, where a higher proportion of maltodextrin reduces the concentration of phenolic compounds in the final powder, as previously reported by Mishra et al. (2014). In contrast, TPC increased with higher feed flow rate and Aonla juice percentage in the blend. This can be attributed to the shorter exposure time of juice droplets to heated air at higher feed flow rates, which minimizes the thermal degradation of phenolic compounds. Additionally, the higher TPC in Aonla juice compared to Giloy juice explains the increased TPC content in powders with a greater proportion of Aonla juice in the blend.

Interestingly, TPC decreased with increasing inlet air temperature up to 170°C, after which TPC showed an increasing trend. This biphasic effect may be due to initial degradation of phenolic compounds at elevated temperatures, followed by thermal‐induced polymerization or synthesis of new polyphenolic structures at higher temperatures. A similar trend was reported by Mishra et al. (2014), where TPC significantly decreased with an increase in inlet air temperature from 125°C to 175°C, but beyond 175°C, the trend reversed.

#### 3.2.4 Powder Recovery

Powder recovery is a crucial parameter for evaluating the efficiency of the spray drying process. The recovery values of the Aonla–Giloy mix powders under different spray drying conditions are presented in Table 4, ranging from 16.59% to 25.81%. ANOVA results (Table 2) revealed that the linear effects of feed flow rate, inlet air temperature, Aonla juice percentage, and maltodextrin concentration, along with the quadratic effect of maltodextrin concentration, had a significant (*p* < 0.05) impact on powder recovery.

Among all process variables, maltodextrin concentration had the most dominant effect on recovery. As a drying aid, maltodextrin encapsulates bioactive compounds, enhancing spray drying efficiency and functional powder properties (Jafari et al. 2017; Kha et al. 2010). Additionally, maltodextrin increases the glass transition temperature of food components, reducing stickiness issues and improving powder yield. Similar results have been reported for Gotu kola powder (Muzaffar et al. 2016; Santhalakshmy et al. 2015; Mishra et al. 2014).

The Aonla juice percentage in the blend also exhibited a positive and significant (*p* < 0.05) effect on powder recovery. As the proportion of Aonla juice increased, the recovery rate improved (Figure 3a). This can be attributed to the higher TSS content of Aonla juice compared to Giloy juice, which reduces water content in the feed, resulting in higher powder yield.

In contrast, higher feed flow rate and inlet air temperature negatively affected powder recovery. The reduction in recovery at elevated feed flow rates may be due to increased wall deposition, as ineffective drying leads to powder adhering to the spray dryer chamber. Similarly, at higher inlet air temperatures, the surface of drying particles may exceed the glass transition temperature, causing the powder to become sticky and deposit on the dryer walls, reducing overall yield. Similar trends have been reported in previous studies (Tonon et al. 2008; Shrestha et al. 2007).

#### 3.2.5 Hygroscopicity

Hygroscopicity is a critical parameter in spray drying, as water absorption can lead to caking, reduced dispersibility, and decreased powder stability. The hygroscopicity of the Aonla–Giloy mix powder under different spray drying conditions is presented in Table 4, with values ranging from 29.39 to 42.16 g/100 g. ANOVA results (Table 2) indicated that feed flow rate, inlet air temperature, Aonla juice percentage, and maltodextrin concentration had significant (*p* < 0.05) linear effects on hygroscopicity. Additionally, interaction effects between inlet air temperature and maltodextrin concentration, along with the quadratic effects of these two variables, were also found to be significant. Among all process variables, inlet air temperature had the highest *F*‐value, suggesting that it had the greatest impact on hygroscopicity.

As shown in Figure 3b, hygroscopicity increased with rising inlet air temperature and Aonla juice percentage in the blend. The increase in hygroscopicity at elevated drying temperatures could be due to the greater water concentration gradient between the powder and the surrounding atmosphere, which accelerates moisture absorption. Similar trends have been reported for spray‐dried rosemary essential oil (Fernandes et al. 2013) and coffee oil (Frascareli et al. 2012).

Aonla juice was found to have a lower MC than Giloy juice. Consequently, as the Aonla juice percentage increased in the blend, the resulting powder had lower MC and greater hygroscopicity.

Conversely, hygroscopicity decreased with increasing feed flow rate and maltodextrin concentration. The lower hygroscopicity at higher feed flow rates may be due to the higher MC in the powder, which reduces the water concentration gradient between the powder and the atmosphere. Similar observations were made in previous studies (Fernandes et al. 2013). Additionally, spray‐dried cactus pear juice powder and betacyanin pigments also exhibited reduced hygroscopicity with increasing maltodextrin concentration (Cai and Corke 2000; Fazaeli et al. 2012). Maltodextrin is a low‐hygroscopicity carrier agent, making it effective in reducing moisture absorption in spray‐dried powders (Tonon et al. 2008; Mishra et al. 2014).

#### 3.2.6 Dispersibility

The dispersibility of the Aonla–Giloy mix powders obtained under different spray drying conditions is presented in Table 5, with values ranging from 75.77% to 91.75%. As shown in Table 2, the linear effects of feed flow rate, inlet air temperature, Aonla juice percentage in the blend, and maltodextrin concentration, along with the quadratic effect of inlet air temperature, had significant (*p* < 0.05) influences on dispersibility. ANOVA results further indicated that maltodextrin concentration had the highest *F*‐value, suggesting it was the dominant factor influencing the dispersibility of the Aonla–Giloy powder in water.

As depicted in Figure 4a, dispersibility decreased with increasing feed flow rate and maltodextrin concentration. At higher feed flow rates, powders retain higher MC due to incomplete drying, which hinders proper dispersion in liquid (Frascareli et al. 2012). Similarly, an increase in maltodextrin concentration reduced dispersibility due to the low hygrophilicity of maltodextrin, making it less effective in promoting powder dissolution (Tonon et al. 2008).

Conversely, dispersibility increased with rising inlet air temperature and a higher proportion of Aonla juice in the blend. The lower MC at elevated inlet temperatures enhances powder flow properties, resulting in improved dispersibility. Additionally, higher Aonla juice content contributed to greater dispersibility, likely due to its higher concentration of water‐soluble solids compared to Giloy juice.

#### 3.2.7 Water Solubility Index

The WSI is an essential reconstitution property used to assess the impact of spray drying parameters on powder functionality (Jafari et al. 2017). The variation in WSI of the Aonla–Giloy mix powder, influenced by different spray drying conditions, is presented in Table 5, with values ranging from 74.63% to 89.78%.

ANOVA results (Table 2) revealed that inlet air temperature, Aonla juice percentage in the blend, and maltodextrin concentration had significant (*p* < 0.05) linear effects on WSI, while maltodextrin concentration also exhibited a significant quadratic effect. Among these factors, maltodextrin concentration had the highest *F*‐value, indicating its dominant role in determining WSI (Table 4).

As shown in Figure 4b, WSI decreased with increasing feed flow rate and maltodextrin concentration. The lower solubility at higher feed flow rates can be attributed to the higher MC in the powder due to ineffective drying, which reduces its ability to dissolve in water (Frascareli et al. 2012). Additionally, higher maltodextrin concentrations reduced WSI, as maltodextrin is low in hygrophilicity, making it less soluble in water (Tonon et al. 2008). Similar trends have been reported for spray‐dried pomegranate juice (Jafari et al. 2017) and guava juice (Patil et al. 2014).

Alternatively, WSI increased with rising inlet air temperature and a higher proportion of Aonla juice in the blend. The higher drying temperature enhances powder solubility by reducing MC and improving surface properties. Similar findings have been reported for Jamun pulp (Santhalakshmy et al. 2015), Jamun juice (Jafari et al. 2017), Guava juice (Patil et al. 2014), and Ginger juice (Phoungchandang and Sertwasana 2010).

#### 3.2.8 Total Color Change (ΔE)

Color is a critical sensory attribute, as it provides consumers with an immediate perception of freshness, flavor, and quality in processed food and beverages. In general, colors closely resembling fresh food are considered more desirable, whereas deviations may indicate either a positive or negative impact on product quality, depending on factors such as natural pigment composition, carrier material concentration, and drying temperature (Patil et al. 2014).

The total color change (ΔE) of the Aonla–Giloy mix powder under different spray drying conditions is presented in Table 5, with values ranging from 13.43 to 18.35. ANOVA results (Table 2) indicated that feed flow rate, inlet air temperature, Aonla juice percentage in the blend, and maltodextrin concentration had significant (*p* < 0.05) linear effects on ΔE, while maltodextrin concentration also exhibited a significant quadratic effect. Among these variables, maltodextrin concentration had the strongest impact, primarily due to its white color, which contributed to an overall lightening effect in the powder (Table 4). A similar trend has been observed for spray‐dried Tucupi (Pires and da Silva Pena 2017) and Pineapple Juice (Jittanit et al. 2010).

As shown in Figure 4c, ΔE was positively correlated with feed flow rate and Aonla juice percentage in the blend. The increase in ΔE with higher aonla juice content is likely due to the darker natural color of Aonla juice, which contains carotenoids and chlorophyll, compared to Giloy juice, which primarily contains chlorophyll. Consequently, as the proportion of Aonla juice increased, the powder color deviated more from its original hue.

In contrast, inlet air temperature exhibited a negative correlation with total color change (ΔE), meaning higher drying temperatures resulted in a reduced ΔE. This could be due to a combined effect of lightening and controlled burning at increased temperatures. The initial color of the Aonla–Giloy juice mixture was greenish‐yellow, which became lighter upon maltodextrin addition. However, at higher inlet air temperatures, slight thermal browning of powder particles may have counteracted the whitening effect, resulting in a final color closer to the original. Similar trends have been documented for spray‐dried passion fruit (Rodríguez‐Hernández et al. 2005) and raspberry powders (Anekella and Orsat 2013).

### 3.3 Sensory Evaluation

The sensory quality of the spray‐dried Aonla–Giloy mix powder juice was evaluated using a 9‐point hedonic scale, assessing appearance, aroma, taste, and OA. The results, presented in Table 5, indicated that the Aonla–Giloy mix powder juice was acceptable across all sensory parameters, with an OA score exceeding 68%.

As shown in Table 2 and Figure 5, the acceptability score significantly (*p* < 0.05) decreased with increasing feed flow rate and maltodextrin concentration. The higher maltodextrin concentration likely led to flavor dilution, as it reduced the relative concentration of the bioactive and flavor compounds present in Aonla and Giloy juices. This effect has been previously observed in spray‐dried pineapple juice, where the addition of maltodextrin reduced sourness and increased sweetness, altering the sensory perception of the final product (Jittanit et al. 2010).

Conversely, the acceptability score significantly (*p* < 0.05) increased with higher inlet air temperature and a greater proportion of Aonla juice in the blend. Aonla juice has a strong, distinct flavor and color, which contributed to a more appealing sensory profile when present in higher proportions. The optimal sensory quality was obtained at a feed flow rate of 2.60 mL/min, an inlet air temperature of 180°C, an Aonla juice proportion of 60.00% in the blend, and a maltodextrin concentration of 22.50%, resulting in an OA score of 87.39%. The lowest acceptability score (68.84%) was recorded at a higher feed flow rate (3.60 mL/min), a lower inlet air temperature (160°C), a lower Aonla juice proportion (40.00%), and a higher maltodextrin concentration (27.50%).

The sensory attributes of the spray‐dried Aonla–Giloy mix powder were significantly influenced by feed flow rate, inlet air temperature, Aonla‐to‐Giloy ratio, and maltodextrin concentration. Optimizing these parameters can enhance consumer acceptance by preserving flavor intensity and ensuring a balanced taste profile.

### 3.4 Optimized and Validation

The results of this study were used to determine the optimal combination of feed flow rate, inlet air temperature, Aonla juice percentage in the blend, and maltodextrin concentration to achieve high‐quality spray‐dried Aonla–Giloy mix powder without compromising its nutritional and functional properties. The optimization process applied specific constraints, aiming to maximize titratable acidity, AA content, TPC, dispersibility, and WSI, while minimizing MC, hygroscopicity, and total color change. A desirability‐driven solution of 0.85 identified the optimal conditions as feed flow rate of 2.70 mL/min, inlet air temperature of 167°C, Aonla juice percentage of 70.00% in the blend, and maltodextrin concentration of 21.50%, resulting in maximum powder recovery (20.31%), AA retention (675.93 mg/100 g), TPC (23,397.43 mg GAE/100 g), dispersibility (89.23%), WSI (90.24%), and OA (86.37%). Additionally, the optimized process conditions minimized MC (6.34%), hygroscopicity (38.26%), and total color change (15.99). The accuracy of the regression models was confirmed through residual analysis, validating their predictive capability for assessing spray‐dried juice quality under varying process conditions (Table 6). Previous studies have demonstrated the effectiveness of spray drying in preserving bioactive compounds in fruit juices, with Mishra et al. (2014) reporting high AA and phenolic content retention in Amla‐based powders. Similarly, Singh et al. (2019) found that optimizing inlet air temperature (175°C–185°C) and maltodextrin concentration (5%–10%) in Jamun pulp powders resulted in higher retention of total phenolic and anthocyanin content, while reducing MC, thereby enhancing shelf stability and functional properties. These findings reinforce the significance of optimizing spray drying parameters such as feed flow rate, inlet air temperature, juice composition, and carrier concentration to improve physical and nutritional quality. The present study aligns with previous research, demonstrating that a desirability‐driven approach can yield high‐quality, nutrient‐rich powders that meet industrial and consumer expectations.

**TABLE 6:** Optimized spray drying conditions and predicted versus experimental values of key quality parameters of Aonla–Giloy mix powder.

Name	Goal	Lower limit	Upper limit	Optimization level	Experimental level
Feed flow rate (mL/min)	In range	2.10	4.10	2.70^a^	2.70^a^
Inlet air temperature (°C)	In range	150	190	167^a^	167^a^
Aonla juice (%) in blended juice	In range	30.00	70.00	70.00^a^	70.00^a^
Maltodextrin concentration (%)	In range	20.00	30.00	21.50^a^	21.50^a^

Response	Goal	Lower limit	Upper limit	Predicted values	Experimental Values
Powder recovery (%)	Maximize	16.59	25.81	20.85^a^	20.31^a^
Moisture content (%)	Minimum	4.58	7.21	6.11^a^	6.34^a^
Ascorbic acid (mg/100 g)	Maximize	377.47	694.61	689.92^a^	675.93^a^
Total phenolic content (mg GAE/100 g)	Maximize	18,325.30	23,436.38	23,653.48^a^	23,397.43^a^
Hygroscopicity (g/100 g)	Minimum	29.39	42.16	39.63^a^	38.26^a^
Dispersibility (%)	Maximize	75.77	91.75	90.58^a^	89.23^a^
Water solubility index (%)	Maximize	74.63	89.78	89.78^a^	90.24^a^
Total color change (ΔE)	Minimize	13.43	18.35	15.53^a^	15.99^a^
Overall acceptability (%)	Maximum	68.84	87.39	87.39^a^	86.37^a^

*Note:* Similar alphabets shows no significant difference at 5% level of significance.

## 4 Conclusion

This study optimized the spray drying conditions for the production of a functional powder from Aonla–Giloy juice blends using RSM. The results demonstrated that process variables, including feed flow rate, inlet air temperature, Aonla juice proportion, and maltodextrin concentration, significantly influenced the physicochemical characteristics, bioactive compound retention, and reconstitution properties of the spray‐dried powder. Increasing the inlet air temperature enhanced powder dispersibility and solubility while reducing MC; however, excessive temperatures led to partial degradation of heat‐sensitive compounds. Maltodextrin effectively functioned as a carrier agent, improving powder recovery and physical stability, although higher concentrations resulted in dilution of bioactive compounds such as AA and phenolics. A higher proportion of Aonla juice contributed to improved retention of bioactive components and enhanced sensory acceptability of the reconstituted beverage. Optimization through RSM identified the most suitable processing conditions as a feed flow rate of 2.70 mL/min, inlet air temperature of 167°C, 70.00% Aonla juice proportion, and 21.50% maltodextrin concentration, resulting in improved solubility, desirable functional attributes, and an overall desirability of 85.36%. The findings highlight the potential of spray drying as an effective technique for producing stable functional powders from herbal–fruit blends. Future research should focus on storage stability, packaging strategies, and scale‐up feasibility to support industrial application of such functional formulations in the food and nutraceutical sectors.

## Author Contributions

**Mohammad Shafiq Alam:** writing – review and editing, validation. **Gurpreet Jhally:** conceptualization, methodology, investigation. **Amit K. Jaiswal:** conceptualization, writing – review and editing, validation. **Sunita Rani:** writing – review and editing, validation. **Prasad Chavan:** software, formal analysis. **Kulwinder Kaur:** writing – original draft, formal analysis, data curation. **Sajeev Rattan Sharma:** conceptualization, writing – review and editing, supervision. **Tarsem Chand Mittal:** resources, project administration.

## Funding

The authors have nothing to report.

## Conflicts of Interest

The authors declare no conflicts of interest.

## Data Availability

The data that support the findings of this study are available from the corresponding author upon reasonable request.
